# Concentration of HSPA1A and transthyretin proteins in the blood serum of patients with bipolar disorder

**DOI:** 10.1192/j.eurpsy.2024.620

**Published:** 2024-08-27

**Authors:** L. Smirnova, E. Epimakhova, A. Seregin, A. Ryzhkova, G. Simutkin

**Affiliations:** ^1^Laboratory of Molecular Genetics and Biochemistry, Mental Health Research Institute, Tomsk National Research Medical Center of the Russian Academy of Sciences; ^2^Biochemistry, Siberian State Medical University; ^3^Affective States Department, Mental Health Research Institute, Tomsk National Research Medical Center of the Russian Academy of Sciences, Tomsk, Russian Federation

## Abstract

**Introduction:**

Insufficient knowledge about the pathophysiological processes in bipolar disorder (BD) leads to difficulties in differentiating this disorder from other affective disorders. Quantitative analysis of serum protein profiles in BD expands our understanding of the pathophysiology of the disease and may aid in subsequent diagnosis. As a result of a previously conducted comparative mass spectrometric study of serum proteins in patients with depression, bipolar disorder and healthy donors, increased expression of Heat Shock 70kDa Protein1A (HSPA1A) and transthyretin was identified.

**Objectives:**

Determination of HSPA1A and transthyretin concentrations in the blood serum of patients with mental disorders.

**Methods:**

Blood serum of 28 patients with bipolar disorder aged 49 years [33;52], 30 patients with recurrent depressive disorder aged 40 years [31; 51] and 130 patients with schizophrenia aged 38 years [31;49], as well as 20 mentally and somatically healthy individuals aged 35 years [31;40] was studied. The amount of Heat shock protein 1A (HSPA1A) and Transthyretin (thyroxine and retinol transport protein) was determined using a Enzyme-linked Immunosorbent Assay Kit from Homo sapiens (Cloud-Clone Corp). Statistical data processing was carried out in the Statistica 12.0 program.

**Results:**

A statistically significant (p = 0.016) increase in the level of HSPA1A was found in patients with BD (0.84 [0.59; 1.09] ng/ml), compared with healthy individuals (0.61 [0.51; 0.77] ng/ml). HSPA1A **p**lays a pivotal role in the protein quality control system, ensuring the correct folding of proteins. It is known that this protein is involved in the embryonic development of the central nervous system, as well as in neuroprotection by preventing the death of neurons due to its anti-apoptotic properties. A statistically significant (p = 0.047) increase in the level of transthyretin was found in patients with BD 21.8 рg/ml, compared with healthy individuals 19.4 pg/ml. Transthyretin plays an important role in ensuring the normal state of the central nervous system and is involved in cognitive processes.

**Conclusions:**

Thus, the HSPA1A and transthyretin are probably involved in the pathogenesis of BD and can be proposed as be proposed as an additional paraclinical criterion for differential diagnosis.

Support by RSF №23-75-00023.

**Disclosure of Interest:**

None Declared

